# Copper Pyrithione Induces Hepatopancreatic Apoptosis and Metabolic Disruption in *Litopenaeus vannamei*: Integrated Transcriptomic, Metabolomic, and Histopathological Analysis

**DOI:** 10.3390/ani15142134

**Published:** 2025-07-18

**Authors:** Jieyu Guo, Yang Yang, Siying Yu, Cairui Jiang, Xianbin Su, Yongfeng Zou, Hui Guo

**Affiliations:** Guangdong Provincial Key Laboratory of Aquatic Animal Disease Control and Healthy Culture, Key Laboratory of Marine Ecology and Aquaculture Environment of Zhanjiang, College of Fisheries, Guangdong Ocean University, Zhanjiang 524025, China; guojieyu888@gmail.com (J.G.); yangyang2002226@126.com (Y.Y.); yu_sying@163.com (S.Y.); 15285189606@stu.gdou.edu.cn (C.J.); suxianbin666@gmail.com (X.S.); zouyongfeng@stu.gdou.edu.cn (Y.Z.)

**Keywords:** CuPT, *Litopenaeus vannamei*, transcriptomics, metabolomics, integrated analysis

## Abstract

Copper pyrithione (CuPT), an emerging biocide used in ship antifouling coatings, may accumulate in marine sediments and pose risks to non-target organisms. However, current research on CuPT toxicity remains limited. *Litopenaeus vannamei*, one of the world’s most important aquaculture shrimp species, relies heavily on its hepatopancreas for energy metabolism, detoxification, and immune responses. Due to their benthic habitat, these shrimps are highly vulnerable to contamination in sediment environments. This study investigated the toxicological response in the hepatopancreas of *L. vannamei* exposed to CuPT (128 μg/L) for 3 and 48 h. Terminal deoxynucleotidyl transferase dUTP Nick-End Labeling (TUNEL) fluorescence staining revealed increased apoptosis, deformation of hepatic tubule lumens, and the loss of stellate structures in the hepatopancreas after CuPT 48 h exposure. A large number of differentially expressed genes (DEGs) were identified by transcriptomics analysis at 3 and 48 h, respectively. Most of these DEGs were related to detoxification, glucose transport, and immunity. Metabolomic analysis identified numerous significantly different metabolites (SDMs) at both 3 and 48 h post-exposure, with most SDMs associated with energy metabolism, fatty acid metabolism, and related pathways. Kyoto Encyclopedia of Genes and Genomes (KEGG) analysis of metabolomics and transcriptome revealed that both DEGs and SDMs were enriched in arachidonic acid metabolism, fatty acid biosynthesis, and glycolysis/gluconeogenesis pathways at 3 h, while at 48 h they were enriched in the starch and sucrose metabolism, amino sugar and nucleotide sugar metabolism, and galactose metabolism pathways. These results suggested that CuPT disrupts the energy and lipid homeostasis of *L. vannamei*. This disruption compelled *L. vannamei* to allocate additional energy toward sustaining basal physiological functions and consequently caused the accumulation of large amounts of reactive oxygen species (ROS) in the body, leading to apoptosis and subsequent tissue damage, and ultimately suppressed the immune system and impaired the health of *L. vannamei*. Our study elucidates the molecular mechanisms of CuPT-induced metabolic disruption and immunotoxicity in *L. vannamei* through integrated multi-omics analyses, providing new insights for ecological risk assessment of this emerging antifoulant.

## Simple Summary

Copper pyrithione (CuPT), an antifoulant used in ship coatings, accumulates in coastal sediments and poses ecotoxicological risks to benthic organisms. This study investigated the molecular response mechanisms in the hepatopancreas of *Litopenaeus vannamei* exposed to CuPT (128 μg/L) for 3 and 48 h. Apoptosis detection, transcriptomic, and metabolomic analysis indicated that prolonged exposure induced hepatopancreatic, apoptosis, impaired energy, and fatty acid metabolism. Concurrently, down-regulation of immune-related genes compromised detoxification and pathogen defense mechanisms, threatening their health and growth. These findings highlight the ecological risks of CuPT to marine crustaceans and provide critical insights into its toxicity mechanisms, offering scientific references for regulating antifouling biocides.

## 1. Introduction

Antifoulants are often added to ship coatings to reduce the adhesion of marine fouling organisms through the continuous leaching out of antifouling chemicals [1]. Organotin compounds, particularly tributyltin (TBT), were the most widely used antifouling agents in the 20th century [2] due to their effectiveness against a wide range of fouling organisms. However, in 2008, the International Maritime Organization officially banned the use of TBT in marine antifouling because of its persistent pollution [3]. Today, metal pyrithiones (MePTs)—a class of organometallic antifoulants including zinc pyrithione (ZnPT) and copper pyrithione (CuPT)—have emerged as an alternative to TBT because of their strong antibacterial and antifungal effects. MePTs are complexes, the most stable of which is CuPT, formed by the binding of two pyridinethione ligands to a central metal cation. According to the statistics of biocide use in antifouling coatings registered worldwide, CuPT constituted 16.7%, and its peak nominal concentration in the registered formulations was 2.9 ± 1.6% *w*/*w* [4]. CuPT has been demonstrated to gradually leach from antifouling coatings [5]. CuPT was initially not classified as a persistent marine pollutant [6], due to its rapid photodegradation under direct light, which converts it to low-toxicity compounds [7]. Its photolytic half-life is estimated to be 7.1 ± 0.2 min under well-illuminated conditions [8]. However, CuPT was capable of accumulating in sediments at very high concentrations and was reportedly detected in sediments from the Pacific coast of Japan and along the north central coast of Vietnam at levels of 22 µg/kg [9] and 420 µg/kg [10], respectively. Furthermore, the low light transmission and high turbidity of certain environments, such as ports and harbors, greatly slow the degradation process of CuPT, making the contaminant persistent [8]. As a consequence, CuPT has emerged as a hazardous contaminant, posing greater risks to benthic crustaceans since it accumulates in the sediments they inhabit.

Crustaceans are extensively utilized as bioindicators of water pollution (such as heavy metals and organic pollutants). Their sensitivity manifested at physiological, behavioral, and molecular levels enables them to play a crucial role in ecosystem health assessments [11]. *Litopenaeus vannamei* currently represents one of the world’s top three aquaculture production species and exhibits high economic value. The adult shrimp are typically found in the muddy seabed, while the juvenile shrimp reside in bait-rich estuaries. They have been employed as model organisms for toxicological studies due to their sensitivity to environmental factors [12]. The hepatopancreas, a vital organ in crustaceans, plays a crucial role in energy metabolism, detoxification, immune response, and stress resistance [13]. Meanwhile, numerous studies have identified the hepatopancreas as a primary target organ for the accumulation and effects of organic pollutants [14,15,16].

Current research on CuPT toxicity in marine crustaceans remains limited, with most studies focusing on only two key aspects. Firstly, the study of the median lethal concentration (LC_50_) of CuPT, such as *Heptacarpus futilirostris* (96 h LC_50_ 2.5 μg/L) [17], *Elasmopus rapax* (96 h LC_50_ 11.5 μg/L) [18], and *Tigriopus japonicus* (96 h LC_50_ 23 μg/L) [19]. Secondly, physiological and biochemical studies have demonstrated that: both RNA/DNA ratio and growth performance were significantly suppressed in *E. rapax* under 2, 5, and 10 μg/L CuPT exposure [18]. The production of reactive oxygen species (ROS) and hepatopancreas apoptotic increased in *L. vannamei* with prolonged 128 μg/L CuPT exposure [20]. Chronic exposure to 1.24 ng/L CuPT induced transgenerational toxicity in *Neomysis awatschensis*, severely compromising larval and adult viability [21]. In the field of molecular biology, there is currently limited research, particularly regarding studies integrating bioinformatics approaches. Our understanding of the specific molecular response mechanisms of the hepatopancreas to CuPT exposure remains scarce.

This study investigated the hepatopancreatic response of *L. vannamei* to CuPT exposure, using dUTP Nick-End Labeling (TUNEL) fluorescence staining to observe apoptosis, non-targeted GC-MS metabolomics to identify significantly different metabolites (SDMs), and transcriptomics to analyze differentially expressed genes (DEGs); finally, the physiological and toxicological responses of the hepatopancreas from *L. vannamei* following CuPT exposure are discussed in an integrated manner, and a connectivity network is mapped based on the association of metabolites with genes. Our findings elucidate the molecular mechanisms underlying the response to CuPT in *L. vannamei* and offer new insights into its effects on crustaceans.

## 2. Materials and Methods

### 2.1. Experimental Design and Sample Collection

The experimental design followed our established protocols as detailed in prior work [20], encompassing all aspects of the study including CuPT solution preparation, shrimp acquisition and acclimation, standardized housing conditions, controlled feeding regimens, and core exposure parameters with specified concentrations, duration periods, and sampling time points.

Briefly, CuPT (97% purity, CAS 14915-37-8) was first dissolved in dimethyl sulfoxide (DMSO, 99.9% purity, CAS 67-68-5) and then diluted in sterilized seawater to achieve a final exposure concentration of 128 µg/L. *L. vannamei* (mean weight: 7.51 ± 1.94 g) were acclimated for 14 days under controlled conditions: salinity 26‰, pH 7.5–7.9, temperature 26.2–26.8 °C, and dissolved oxygen 5.8–6.2 mg/L. Following acclimation, shrimp were allocated to two groups (three biological replicates per group)—control group (C, n = 30 per biological replicate) and CuPT-exposed (T, 128 μg/L, n = 30 per biological replicate)—and exposed for 48 h. The test solution was renewed every 24 h. Feeding was suspended 24 h prior to exposure and throughout the experimental period. Both control and treatment groups were maintained under identical acclimation conditions.

Importantly, based on robust evidence from our prior work [20] demonstrating that DMSO at 0.128 mL/L (maximum solvent concentration used herein) did not induce physiological, metabolic, or transcriptomic alterations in *L. vannamei* hepatopancreas, the current experimental design omitted a separate DMSO control group. All comparisons were thus made relative to a no-solvent control.

Shrimp in the control group were maintained under identical conditions for the full 48 h but were only sampled at 0 h (C_0; n = 12) to establish baseline data. The exposed group was sampled at 3 h (T_3; n = 12) and 48 h (T_48; n = 12). For each group, hepatopancreas from 3 shrimp were fixed in 4% paraformaldehyde for a TUNEL assay, while tissue from the remaining 9 shrimp was flash-frozen in liquid nitrogen for transcriptomic and metabolomic analyses.

### 2.2. TUNEL Analysis for Hepatopancreas Cell Apoptosis

Hepatopancreas tissues were fixed in 4% paraformaldehyde, dehydrated, embedded in paraffin, and sectioned at 4 μm. Following deparaffinization, the sections underwent TUNEL staining using a commercial kit (Wuhan Servicebio Biotechnology Co., Ltd., Wuhan, China) strictly adhering to the manufacturer’s instructions. The observations were conducted using a Nikon Eclipse C1 orthogonal fluorescence microscope (Nikon, Tokyo, Japan). Additionally, the imaging system Nikon DS-U3 (Nikon, Tokyo, Japan) was employed to capture images of the specimens.

### 2.3. Transcriptome Sequencing, Analysis, and Validation

#### 2.3.1. RNA Extraction, Library Construction, and RNA Sequencing

Total RNA was extracted from hepatopancreas tissues of each sample using Trizol reagent (Invitrogen, Carlsbad, CA, USA) following the manufacturer’s instructions. The concentrations and qualities of the RNA were quantified and verified using a spectrophotometer (NanoDrop Technologies, Wilmington, DE, USA) and agarose gel electrophoresis, respectively.

The cDNA libraries were prepared using Illumina’s NEBNext^®^ Ultra™ RNA Library Prep Kit (Illumina Inc., San Diego, CA, USA). Briefly, the first strand of cDNA was synthesized using fragmented mRNA as a template and random oligonucleotides as primers. The second strand cDNA synthesis was subsequently performed using DNA Polymerase I and RNase H. The purified cDNA fragments were end repaired, an A-tailed added, and ligated to Illumina sequencing adapters. The ligation reaction was purified with AMPure XP Beads (1.0×). cDNA libraries were quantified by Agilent high-sensitivity DNA analyzer on the bioanalyzer 2100 (Agilent, Singapore). Sequencing was conducted using the Illumina HiSeq™ 2500 platform (Illumina Inc., San Diego, CA, USA).

The raw data were filtered using Fastp (v0.19.3) to remove reads with adapters, reads with N content exceeding 10% of bases, and reads with low-quality bases (Q ≤ 20) exceeding 50% of bases [22,23]. This produced clean reads, which were used for subsequent analyses.

#### 2.3.2. Alignment to Reference Genome

HISAT (v2.1.0) was utilized to construct the index, with the Clean Reads subsequently aligned with the reference genome [24,25]. FeatureCounts (v1.6.2) was employed to compute the gene alignment [26], with Fragments Per Kilobase of Transcript per Million fragments mapped (FPKM) values calculated for each gene based on its length. The FPKM was then employed as an indicator of the level of gene expression.

#### 2.3.3. Identification of DEGs and Functional Enrichment Analysis

DESeq2 (v1.22.1) software was employed to identify the expression differences between CuPT-exposed and control groups [27]. The Benjamini–Hochberg method was used to assess the false discovery rate (FDR) during multiple hypothesis testing. DEGs were then identified on the basis of a |log_2_Fold Change| ≥ 1 with FDR < 0.05. The screened DEGs were subjected to Kyoto Encyclopedia of Genes and Genomes (KEGG) [28] and Gene Ontology (GO) enrichment analysis. We used R software (v4.3.1) [29] for statistical analyses and visualization.

#### 2.3.4. Validation of RNA-Seq Results with Quantitative Real-Time PCR (q-PCR)

To validate transcriptome findings, 14 differentially expressed genes (DEGs) were selected for q-PCR analysis using β-actin as an internal reference gene. Gene-specific primers (designed with Primer Premier 5.0) are listed in Table 1. cDNA synthesis was performed using the PrimeScript™ RT reagent Kit (Takara Bio, San Jose, CA, USA). q-PCR was conducted with ChamQ Universal SYBR qPCR Master Mix (Vazyme, Nanjing, China), with the following conditions: 95 °C for 6 min, followed by 40 cycles of 95 °C for 5 s, 60 °C for 15 s, and 72 °C for 10 s. The relative gene expression levels of the chosen genes were calculated using the 2^−ΔΔCt^ method [30].

### 2.4. Metabolite Extraction, Detection, and Analysis

#### 2.4.1. Metabolite Extraction and Detection

The hepatopancreatic tissues were thawed on ice. Cold steel balls were added to approximately 50 mg of one sample, and this underwent homogenization at 30 Hz for 3 min. A total of 1 mL of ice-cold 70% methanol, containing a suitable internal standard mix, was added to the homogenized sample, which was then vigorously mixed for 5 min. Subsequently, the mixture was subjected to centrifugation at 12,000 rpm at 4 °C for 10 min. Following centrifugation, 400 µL of the supernatant was transferred to a new EP tube and stored in a refrigerator at −20 °C overnight. The next day, the sample was centrifuged at 12,000 rpm at 4 °C for 3 min. Subsequently, 200 µL of the supernatant was transferred to an injection vial for GC-MS analysis.

#### 2.4.2. Metabolite Analysis and Identification

Qualitative analysis was performed based on the retention time of detected substances (RT), precursor/product ion pairs, and comparison secondary spectral data obtained from the MWDB (Wuhan Metwell Biotechnology Co., Ltd., Wuhan, China). Quantitative analysis employed the multiple-reaction monitoring (MRM) mode of triple quadrupole mass spectrometry (TQMS). The mass spectrometry data underwent processing using Analyst (v1.6.3) software, while MultiQuant was used to integrate and correct the chromatographic peaks.

Unsupervised principal component analysis (PCA) was conducted within the statistical R software (v4.3.1), utilizing the prcomp function. The R package MetaboAnalystR 3.0 was employed to generate variable importance in projection (VIP) scores from the Orthogonal partial least squares-discriminant analysis (OPLS-DA) model. The screening criteria of SDMs was VIP ≥ 1 with |log_2_Fold Change| ≥ 1.

Identified SDMs were annotated using the KEGG database. Significantly enriched pathways were identified using a hypergeometric test’s *p*-value (*p* < 0.05 considered statistically significant).

### 2.5. Integrative Analysis of Metabolome and Transcriptome

Correlation analyses were conducted on the genes and metabolites observed in each distinct group. The Pearson correlation coefficients for the genes and metabolites were calculated using the cor program in R. A nine-quadrant plot was created to illustrate the multiple differences in metabolites for genes with Pearson correlation coefficients above 0.8 in each distinct group. Additionally, the DEGs and SDMs of the same subgroupings were concurrently mapped onto KEGG pathway diagrams and illustrated with bar charts based on the results of the differential metabolite and transcriptome analysis, respectively.

## 3. Results

### 3.1. Apoptosis of Hepatopancreatic Cells After CuPT Exposure

**Figure 1:**
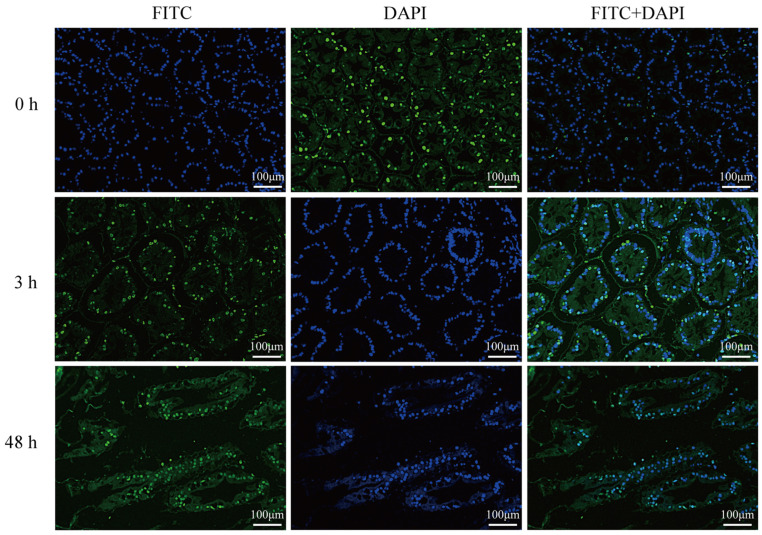
Histological analysis of hepatopancreas tissue in *L. vannamei* exposed to 128 μg/L CuPT for 0, 3, and 48 h (positive apoptotic cell nuclei are colored in green).

TUNEL-FITC/DAPI co-staining of hepatopancreas sections revealed apoptosis and structural disintegration in *L. vannamei* exposed to 128 µg/L CuPT (Figure 1). At 0 h post-exposure, merged images exhibited sparse green fluorescence with minimal co-localization, in contrast to uniformly distributed blue nuclei, indicating limited baseline apoptosis. After 3 h of exposure, discrete co-localized foci appeared, signifying the initiation of apoptosis. By 48 h, increased areas exhibited blue-green co-localization, indicating extensive apoptosis. Additionally, compared to 0 h and 3 h, hepatic tubules displayed lumen deformation and distinct loss of stellate structure after 48 h of exposure.

### 3.2. Transcriptome Sequencing Results and Quality Analysis

A total of nine samples was subjected to transcriptome sequencing analysis in this experiment. Following the removal of all low-quality reads, connector contamination, and reads with a high unknown base N content, a total of 65.79 Gb of clean data was obtained (Table S1).

The alignment results of the transcriptome sequencing data to the reference genome are presented in Table S2. For all sequenced samples, more than 89% of reads were aligned to the reference genome, and more than 70% of reads were uniquely aligned to specific genomic locations. These data demonstrated that good sequencing quality could be used for subsequent analysis.

### 3.3. Transcriptome Analysis of L. vannamei After CuPT Exposure

**Figure 2:**
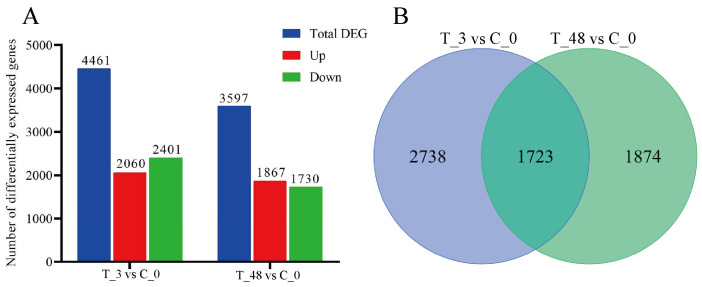
Number of DEGs (**A**) and Venn diagram of DEGs in different groups (**B**).

A total of 4461 (2060 up-regulated and 2401 down-regulated) and 3597 (1867 up-regulated and 1730 down-regulated) DEGs were identified in T_3 and T_48 post-exposure in comparison to C_0, respectively; furthermore, 1723 DEGs were shared both in T_3 and T_48 (Figure 2B), and the results are presented in Figure 2A.

**Figure 3:**
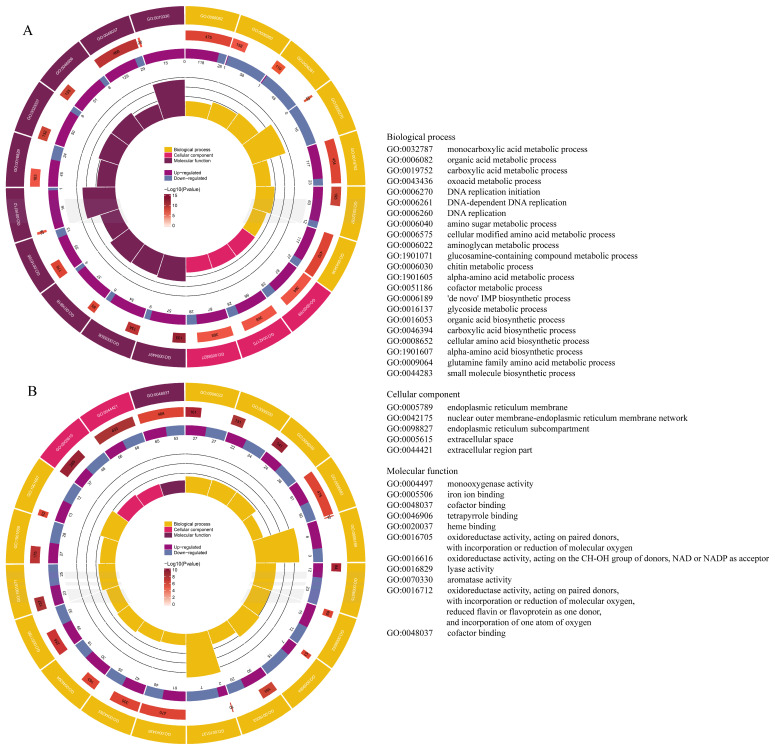
Gene Ontology assignments of DEGs in T_3 vs. C_0 (**A**) and T_48 vs. C_0 (**B**) comparisons.

To reveal the biological function of these DEGs, GO and KEGG analysis were conducted. For GO analysis, all DEGs were annotated into the GO database and subsequently classified into three categories: Biological Process (BP), Cellular Component (CC), and Molecular Function (MF). In the enrichment analysis, the 50 GO terms with the smallest *p*-values were selected to be shown in Figure 3. The greatest number of DEGs were found in the BP category in both T_3 and T_48. Including carboxylic acid metabolic process (GO:0019752), organic acid biosynthetic process (GO:0016053), oxoacid metabolic process (GO:0043436), and so on. In the CC category, the DEGs were primarily enriched within intracellular locations, particularly within the endoplasmic reticulum membrane (GO:0005789) and endoplasmic reticulum subcompartment (GO:0098827) in T_3, while the DEGs were predominantly concentrated to extracellular locations, such as the extracellular space (GO:0005615) and extracellular region part (GO:0044421) in T_48. In the MF category, DEGs were mainly enriched in cofactor bounding (GO:0048037).

**Figure 4:**
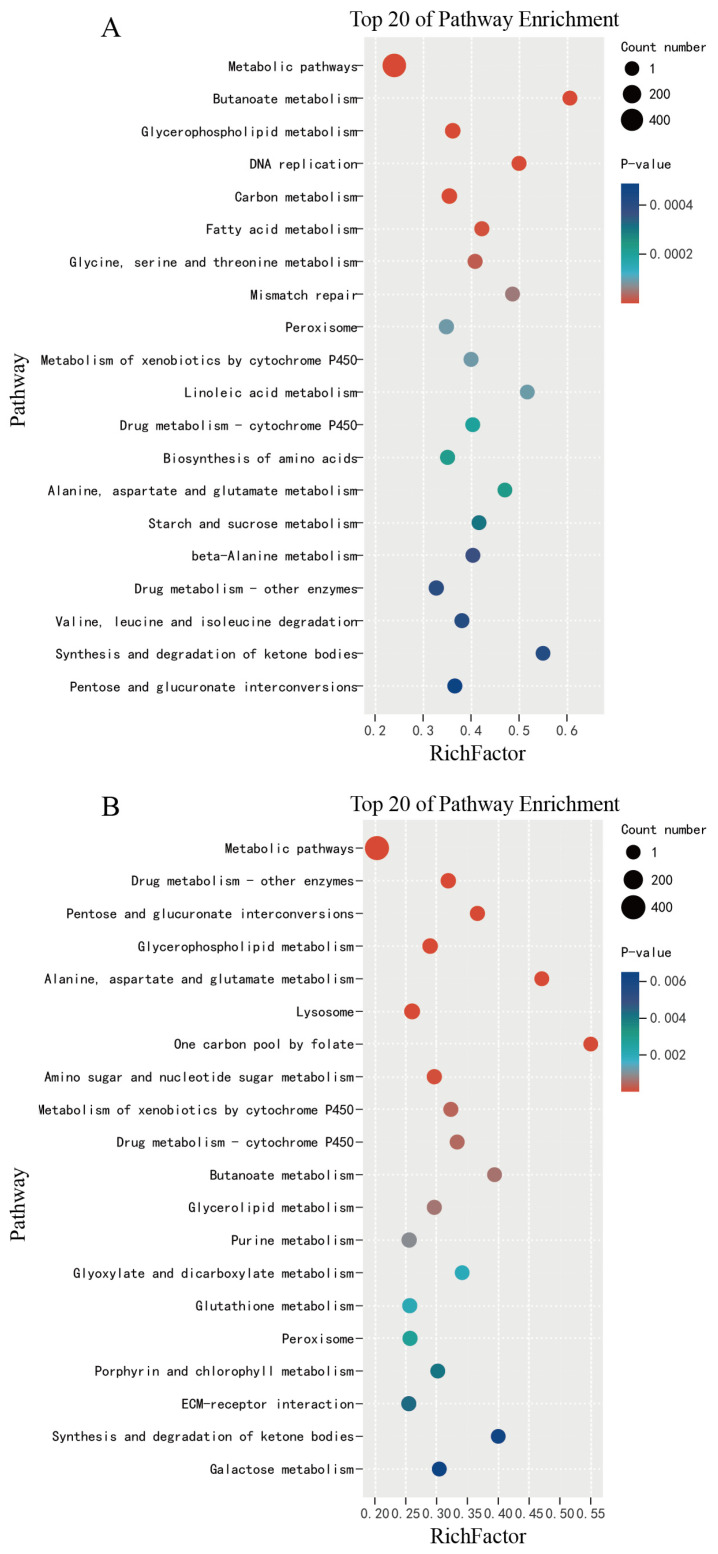
A scatter plot of KEGG enrichment for DEGs in T_3 vs. C_0 (**A**) and T_48 vs. C_0 (**B**) comparisons.

Furthermore, all DEGs were subjected to KEGG analysis, enriched to a total of 250 pathways. The top 20 pathways are presented in Figure 4. Compared to C_0, the most DEGs were found to be enriched in metabolic pathways (ko01100) in both T_3 and T_48. In addition, the pathways for the metabolism of xenobiotics by cytochrome P450 (ko00980), drug metabolism–cytochrome P450 (ko00982), carbon metabolism (ko01200), glycerophospholipid metabolism (ko00564), lysosome (ko04142), glutathione metabolism (ko00480), and peroxisomes (ko04146) were significantly enriched at both T_3 and T_48.

**Figure 5:**
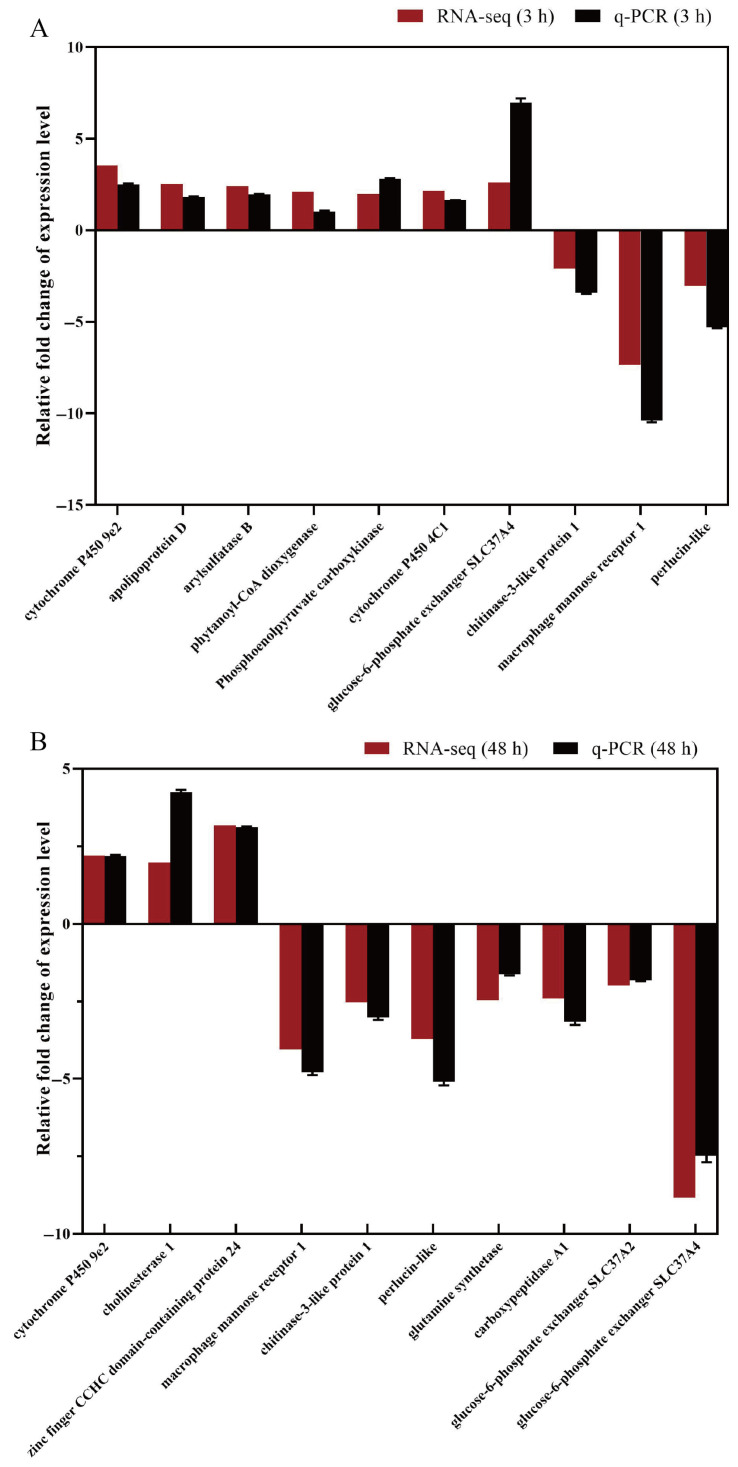
Comparison of gene expression data between RNA-Seq and q-PCR. T_3 (**A**), T_48 (**B**).

The results of the DEGs verification are shown in Figure 5. The results of the q-PCR were in good agreement with the results of the transcriptome sequencing. This proves that the results of the transcriptome sequencing were reliable.

### 3.4. Metabolomic Analysis of L. vannamei After CuPT Exposed

**Figure 6:**
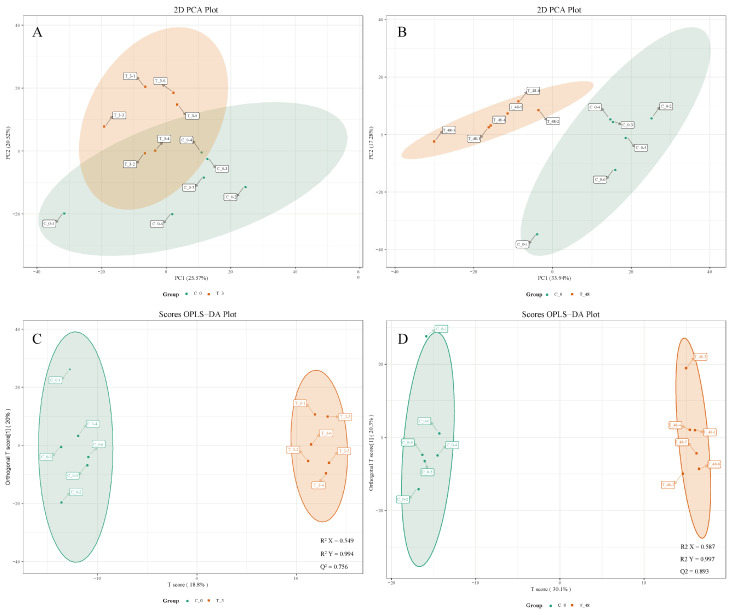
PCA score plot for T_3 vs. C_0 (**A**) and T_48 vs. C_0 (**B**). OPLS-DA score plot for T_3 vs. C_0 (**C**) and T_48 vs. C_0 (**D**).

A total of 891 different metabolites were identified, including amino acids, fatty acyl groups, lipids, peptides, acylcarnitines, nucleic acids, and organic acids. The results of PCA are shown in Figure 6. Samples from T_3 were concentrated in the upper left quadrant, while those from C_0 were concentrated in the lower right. The first principal component (PC1) and the second principal component (PC2) collectively accounted for a total of 46.09%, with PC1 accounting for 25.57% and PC2 accounting for 20.55%. Samples in the T_48 were concentrated on the left side, while samples in the C_0 were concentrated on the right side. PC1 and PC2 together accounted for 51.22% of the total, of which PC1 accounted for 33.94% and PC2 17.28%.

The metabolome data were analyzed according to the OPLS-DA model, and the scores for each group were plotted [31] (Figure 6). Similarly to PCA analyses, a clear separation in metabolic profiles was evident when comparing the T_3, T_48, and C_0. Meanwhile, based on the OPLS-DA results, 49 and 164 SDMs were identified in T_3 and T_48, respectively.

**Figure 7:**
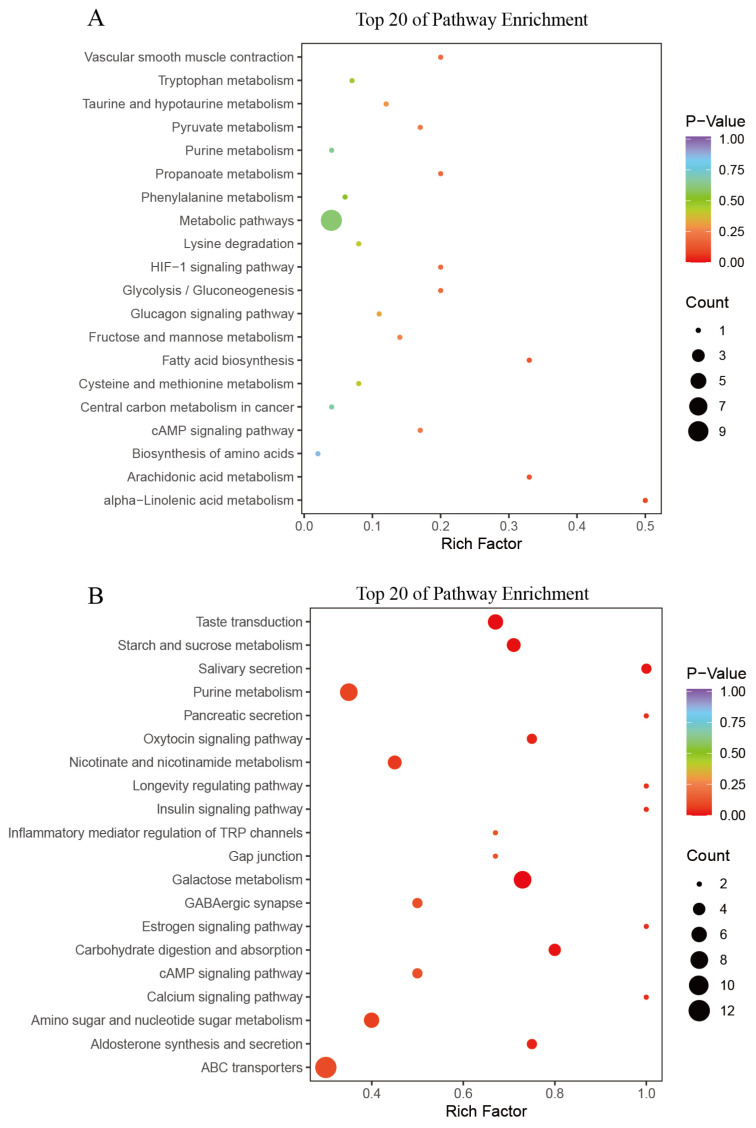
A scatter plot of KEGG enrichment for SDMs in T_3 vs. C_0 (**A**) and T_48 vs. C_0 (**B**) comparisons.

The SDMs were subjected to further analysis via the KEGG database, with the results presented in Figure 7. A total of 49 SDMs mainly enriched in metabolic pathways, including those associated with alpha-linolenic acid metabolism (ko00592), fatty acid biosynthesis (ko00061), arachidonic acid metabolism (ko00590), and glycolysis/gluconeogenesis (ko00010) in the T_3 vs. C_0 comparison. A total of 164 SDMs were primarily associated with starch and sucrose metabolism (ko00500), purine metabolism (ko00230), nicotinate and nicotinamide metabolism (ko00760), galactose metabolism (ko00052), amino sugar and nucleotide sugar metabolism (ko00520), and ABC transporters (ko02010), as well as a range of additional pathways in the T_48 vs. C_0 comparison.

### 3.5. Integrated Analysis of Metabolome and Transcriptome

**Figure 8:**
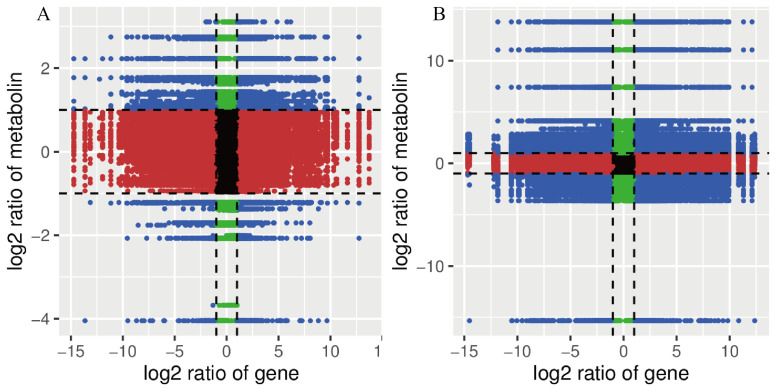
Nine-quadrant associate analysis of DEGs and SDMs in T_3 vs. C_0 (**A**) and T_48 vs. C_0 (**B**) comparisons.

**Figure 9:**
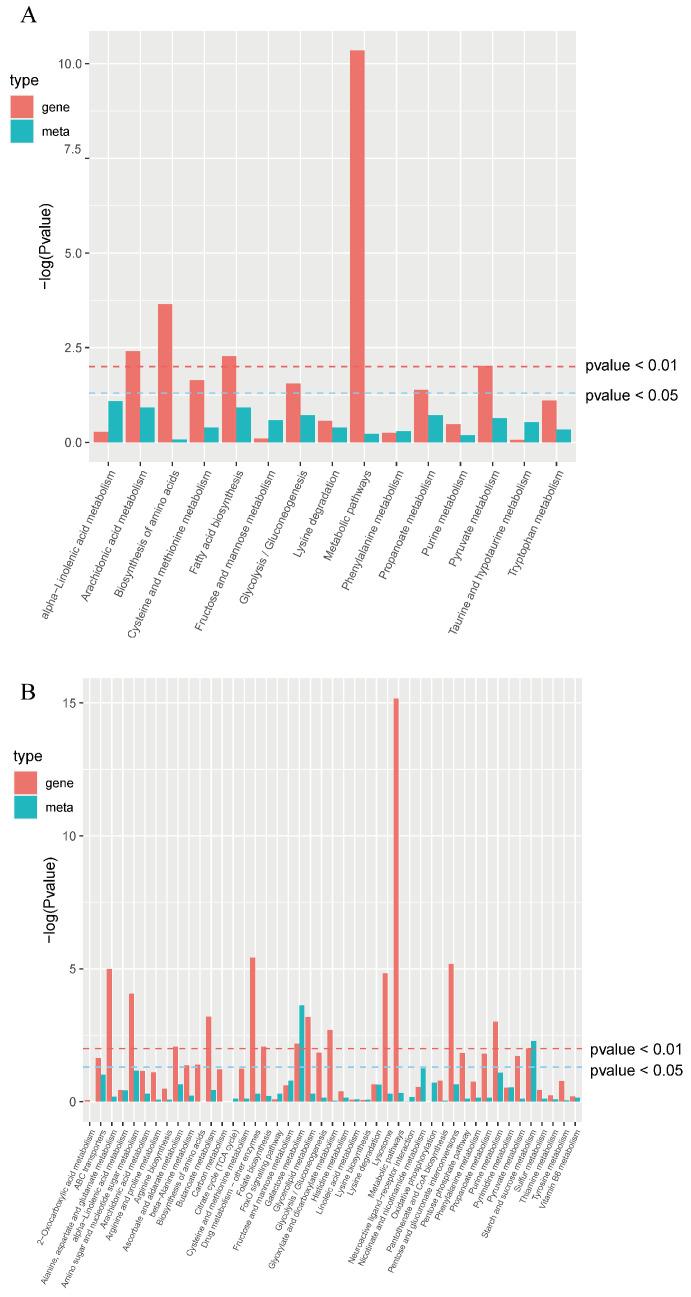
Enrichment pathways of SDMs and DEGs in T_3 vs. C_0 (**A**) and T_48 vs. C_0 (**B**) comparisons.

An integrated analysis was performed on the results of the transcriptomics and metabolomics. A nine-quadrant plot (Figure 8) illustrates the alterations observed in all DEGs and SDMs in the comparison of T_3 vs. C_0 and T_48 vs. C_0. There was a positive correlation between the DEGs and SDMs in Quadrants 3 and 7. In contrast, there was a negative correlation between the two in Quadrants 1 and 9. In T_3 vs. C_0, there were more DEGs and SDMs in Quadrants 4 and 6, indicating a large number of genes and metabolites whose expression was not synchronized, and in T_48, there were fewer genes and metabolites in Quadrants 4 and 6, suggesting that the expression of the genes and metabolites was converging There were markedly greater numbers of DEGs and SDMs in *L. vannamei* in T_48, in comparison to C_0. The DEGs and SDMs were significantly enriched in the glycolysis/gluconeogenesis, fatty acid biosynthesis, and arachidonic acid metabolism pathways in the T_3 vs. C_0. The DEGs and SDMs in the T_48 vs. C_0 were found to be significantly enriched in the pathways of starch and sucrose metabolism, amino sugar and nucleotide sugar metabolism, and galactose metabolism (Figure 9).

## 4. Discussion

### 4.1. CuPT Exposure Affects Energy Metabolism

Studies have shown that shrimp predominantly utilize carbohydrate and triglycerides as their main energy sources during locomotion, with minimal protein catabolism. For instance, *Penaeus japonicus* primarily relies on carbohydrate metabolism to meet energy demands during swimming and escape responses [32]. Similarly the *L. vannamei* primarily utilizes glycogen as its energy source during the act of bouncing; it also utilizes a combination of glycogen and triglycerides for energy during swimming [33]. Glycolysis/gluconeogenesis is a crucial metabolic pathway, wherein glucose can be regenerated from lactate, pyruvate, and amino acids, among other substrates, during gluconeogenesis. It has been demonstrated that both glycolysis and gluconeogenesis-related genes were significantly up-regulated in the hepatopancreas of the *L. vannamei* under hypoxic stress [34]; similar results were observed under high alkalinity stress [35]. In addition, a study on *Chanos chanos*, under low-temperature stress found that lactate content changed significantly, highlighting that metabolic strategies involving lactic acid significantly influence the stress resistance of organisms [36]. Our multi-omics analysis revealed significant enrichment of DEGs and SDMs in glycolysis/gluconeogenesis pathways within *L. vannamei* hepatopancreas in T_3 and T_48. Notably, the level of lactate in *L. vannamei* was markedly diminished. This phenomenon might be attributed to the increased energy required to maintain physiological homeostasis in shrimp under the influence of CuPT exposure, leading to lactate being converted to glucose and glycogen through gluconeogenesis, thereby stimulating the glycolysis and gluconeogenesis in the hepatopancreas.

The solute carrier (SLC) transporter family, one of the largest families of membrane proteins, is a crucial component of cellular membranes [37]. These transporters mediate the transmembrane movement of diverse substrates, including carbohydrates, amino acids, inorganic ions, neurotransmitters, and small organic molecules. The SLC37 family is localized in the endoplasmic reticulum membrane and contains four sugar phosphate exchangers, A1, A2, A3, and A4, which are essential for the maintenance of glucose homeostasis in organisms [38]. It has been reported that the genes of the SLC37 family were up-regulated in low-salinity environments, while they were down-regulated in high-salinity environments in *Oratosquilla oratoria* under different salinity stresses [39]. SLC37A4 is a ubiquitously expressed transporter responsible for glucose-6-phosphate (G6P) translocation across the endoplasmic reticulum membrane. During glycogenolysis, cytoplasmic G6P is transported into the endoplasmic reticulum via SLC37A4, where it undergoes dephosphorylation to glucose [40]. SLC37A2, another member of the SLC37 family, is also involved in G6P transport and phosphate ion transmembrane transport. The expression of the *slc37a2* remained at a relatively stable level in *Procambarus clarkii* under low-salinity environments, while it exhibited a sustained high level under high-salinity conditions, potentially mitigating stress through enhanced carbohydrate transport [41]. Our data clearly demonstrate that the expression of SLC37 genes in *L. vannamei* shows significant temporal variation following CuPT exposure; *slc37a4* transcription surges in T_3, while both *slc37a4* and *slc37a2* are suppressed in T_48. This may indicate that during the initial phase of exposure, *L. vannamei* attempted to maintain intracellular glucose homeostasis by increasing the expression of *slc37a4*, but with prolonged exposure, cellular damage occurred, leading to a decline in the expression of sugar transport-related genes, which impaired cellular energy metabolism.

It is well-established that organisms increase their metabolic rate in response to challenges in order to meet additional energy demands. Metabolomic analysis of *Symphysodon aequifasciatus* subjected to cold stress in fish studies reveals a significant activation of starch and sucrose metabolic pathways, indicating the utilization of these pathways as an adaptive strategy to meet the additional energy demands associated with the stressor [42]. Similarly, bisphenol A exposure disrupts starch–sucrose metabolic flux in *Danio rerio*, underscoring the vulnerability of carbohydrate homeostasis to xenobiotic stress [43]. Significant effects on the hepatopancreatic starch and sucrose metabolic pathways were also observed in *L. vannamei* exposed to the marine antifouling agent DCOIT [44]. Energy metabolism of *Penaeus monodon* [45] and *L. vannamei* [46] was significantly affected under ammonia and nitrite exposure, respectively. A metabolomics study of *Macrobrachium rosenbergii* exposed to ammonia stress revealed that SDMs were significantly enriched in amino sugar and nucleotide sugar metabolism pathways, suggesting the consumption of large amounts of sugars to provide energy [47]. Our study revealed that the genes and metabolites related to starch and sucrose metabolism, amino sugar and nucleotide sugar metabolism, and galactose metabolism were significantly enriched in *L. vannamei* after 48 h of CuPT exposure, suggesting that the *L. vannamei* attempted to maintain normal physiological activities by mobilizing additional energy reserves.

### 4.2. CuPT Exposure Affects Lipid Metabolism

Arachidonic acid (ARA), an ω-6 polyunsaturated fatty acid, functions as a pivotal bioactive lipid mediator in vertebrates and invertebrates. While implicated in inflammatory pathologies and carcinogenesis, ARA also exerts essential immunomodulatory effects through its oxygenated metabolites [48]. The SDMs of *L. vannamei* exhibited a notable enrichment in the ARA pathway following microcystin exposure, particularly in hepatopancreatic tissues [49]. In aquatic animals, ARA serves to mitigate the effects of osmotic pressure [50]. The addition of ARA to the diet of crustaceans has been demonstrated to inhibit inflammation and enhance antioxidant properties [51]. In BaCl2 exposure experiments with *Ruditapes decussatus*, a significant increase in ARA was observed [52]. Addition of histamine to the diet of *Anguilla rostrata* has been reported to result in significant down-regulation of 20-hydroxy-5 Z,8 Z,11 Z,14 Z-eicosatetraenoic acid (20-HETE), a metabolite associated with arachidonic acid metabolism, in the liver, which may interfere with normal liver function and reduce its resistance to stress and disease [51]. Our multi-omics analysis revealed coordinated activation of cyclooxygenase (COX) and cytochrome P450 (CYP450) branches within the arachidonic acid metabolic network (ko00590) in *L. vannamei*, suggesting dual roles in immunoregulation and energy homeostasis. In T_3, thromboxane-A synthase (TBXAS1) and prostaglandin F2alpha synthase (PRXL2B) exhibited significant up-regulation, with their principal metabolites, including prostaglandins (PGs) and thromboxanes (TXs), playing a pivotal role in regulating the pro-inflammatory response of the body [53]. In addition, the metabolite 20-HETE content decreased significantly in T_3, suggesting that CuPT may interfere with fatty acid metabolism in shrimp at the early stage of exposure, which may have an impact on immunity. It has been demonstrated that a notable elevation in secretory phospholipase A2 (PLA2G) activity was observed in *Limnoperna fortunei* after zinc oxide nanoparticle stress, accompanied by an increased production of ARA [54]. A comparable phenomenon was observed in the present experiment, where PLA2G was significantly up-regulated in both T_3 and T_48. This suggested that PLA2G played an important regulatory role in maintaining the relative stability of ARA throughout the entire period of stress, and *L. vannamei* may maintain normal fatty acid metabolism through this mechanism.

### 4.3. CuPT Exposure Affects Detoxification and Immunity

The cytochrome P450 (CYP) superfamily, ubiquitously expressed across phylogenetic lineages, orchestrates the anabolic/catabolic reactions of endogenous compounds (e.g., steroids, fatty acids) and xenobiotic biotransformation. Critically, CYPs mediate metabolic activation of pro-toxins while executing phase I detoxification through hydroxylation and epoxidation [55]. Transcriptomic evidence reveals CYP pathway enrichment (*p* < 0.001) in *L. vannamei* hepatopancreas under diverse stressors, including pathogen challenge and chemical exposure [56]. A significant up-regulation of the *cyp4c1* and *cyp9e2* genes was detected in the analysis of insecticide resistance in Aedes aegypti [57]. The expression levels of *cyp4c1* and *cp2l1* genes were differential changed in the *L. vannamei* following white spot syndrome virus (WSSV) infection [58]. In our study, KEGG enrichment analysis revealed that the DEGs were significantly enriched in the metabolism of the xenobiotics by cytochrome P450 (ko00980), and the drug metabolism–cytochrome P450 (ko00982) pathway in T_3 and T_48, the gene expression of *cp4c1* and *cp9e2* was significantly up-regulated upon CuPT exposure. This suggests that CuPT is transformed via CYP-related pathways (evidenced by 20-HETE depletion and ko00980 enrichment), constituting the primary detoxification mechanism in *L. vannamei*.

Chitin and C-type lectin play significant roles in the innate immune response of crustaceans, promoting agglutination, phagocytosis, and encapsulation and exhibiting bacteriostatic and antiviral activities [59]. The chitinase-like protein CHIT3L1 facilitates chitin turnover, while C-type lectin members like macrophage mannose receptor 1 (MCR1) and perlucin orchestrate pathogen opsonization via terminal mannose/galactose recognition. A recent study indicated that both *chi3l1* and *perlucin-like* genes were significantly down-regulated in *L. vannamei* following WSSV infection [58]. In our study, the expression levels of chi3l1, mrc1, and perlucin-like genes in *L. vannamei* were significantly down-regulated during CuPT exposure. This transcriptional suppression likely disrupts chitin-mediated pathogen surveillance and lectin-dependent opsonization, potentially rendering *L. vannamei* immunocompromised.

**Figure 10:**
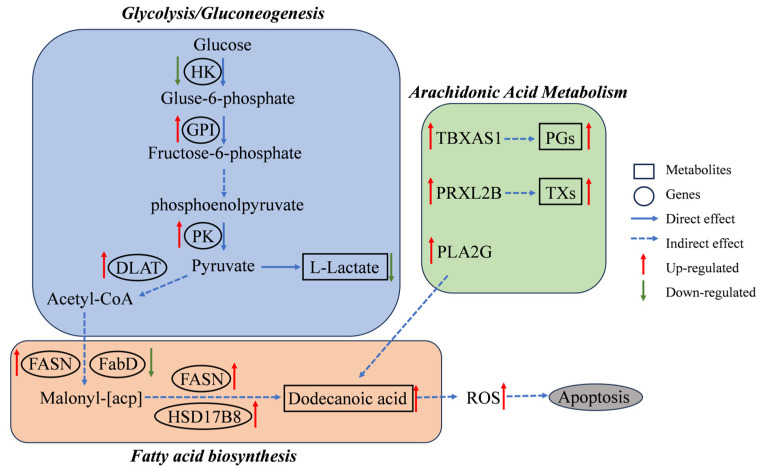
Schematic diagram summarizing the proposed mechanisms of CuPT toxicity in *L. vannamei* hepatopancreas based on integrated transcriptomic and metabolomic analyses. CuPT exposure disrupts energy metabolism (glycolysis/gluconeogenesis, starch/sucrose metabolism) and lipid metabolism (fatty acid biosynthesis, arachidonic acid metabolism), leading to increased energy demand, ROS accumulation, apoptosis, tissue damage, and immune suppression (down-regulation of detoxification CYPs, chitinase/lectin genes).

In summary, combining the results of transcriptomics and metabolomics, we suggested that CuPT mainly causes serious effects on energy metabolism and fatty acid metabolism of *L. vannamei*, which in turn causes disruption of immune function and cellular homeostasis and ultimately results in tissue damage; a simple schematic diagram is shown in Figure 10.

## 5. Conclusions

In this study, we conducted integrated transcriptomic and metabolomic analyses to investigate the effects of CuPT exposure on the genes and metabolites in *L. vannamei* hepatopancreas. Our findings demonstrate that copper pyrithione disrupts energy metabolism, lipid homeostasis, and immune function in *L. vannamei* at sublethal concentrations (128 μg/L). This supports stricter regulation of CuPT in antifouling paints, such as concentration caps in coastal zones or substitution with less persistent alternatives. We recommend monitoring sediments in high-risk areas (e.g., ports) to mitigate ecological risks. This study highlights the toxicity of CuPT to non-target organisms and underscores the potential environmental risk posed by this compound to aquatic species. Its continued accumulation in sediments could pose a significant threat to benthic organisms or their predators. Our findings provide a foundation for further research on the toxicity mechanism of CuPT and the support of regulatory assessments.

## Figures and Tables

**Table 1:** Primers used for q-PCR.

Gene Name	Genebank ID	Sequences (5′-3′)
*chi3l1*	XM_027369287.1	F:ACTGCTCGTCCTACTTGCAC
		R:GTCGAATTTGCCATCGCCAG
*perlucin-like*	XM_027364808.1	F:CACCCAGGAACGTCTATGCT
		R:TATCAGGCATCCCGTTAGCC
*mrc1*	XM_027372201.1	F:CGCCAACGCAGTTTAAGGAAG
		R:TGTTCTCCTCCGTGTTCGTT
*arsb*	XM_027370159.1	F:GAGTTACGTGCAGCCTCTGT
		R:CCGATCATGTGCGTGGAGTA
*apod*	XM_027367111.1	F: CACGGTCGCAGTTCACAAC
		R:CATAGTCGGTGTCCAGCACG
*cyp9e2*	XM_027375382.1	F:GCCTCGGGTCTGAAAGTCTG
		R:TGACCGATGAATGGGACCAC
*cyp4c1*	XM_027375771.1	F:TTCGGTTCTCGCCGTATCAG
		R:GATCATGCACGTCGGGAGAA
*zcchc3*	XM_027361571.1	F:AGCGGTGTAAATCCTGCAAC
		R:TTTTGACGCTGAAGTTGGCTG
*slc37a2*	XM_027373750.1	F:TACCACCTCTCAAGGAAGCC
		R:CTAGGAGTGTTGCGGAGTTT
*slc37a4*	XM_027355483.1	F:ACCAACATGACTGTGAGGGAC
		R:TGAAGCCACCCGTTTCTATGG
*bche*	XM_027371209.1	F:CGCCCTTATCTGCACCACTT
		R:GACACCAGGAACCGCATCTC
*Pck1*	XM_027371589.1	F:CTTCCATCTCCGTCACCCAT
		R:AGTTGCTGTACTTGGGCAGG
*phyh*	XM_027363522.1	F:GGGGAACGACACAGGAAAAC
		R:CAGGCTGTCCTCTACGCCTC
*glul*	XM_027354704.1	F:GCTCCAAGACACGTACCCTC
		R:CTTGTAGGTCTCGCAGAGCA
*β-actin*	AF300705	F:CATCAAGGAGAAACTGTGCTACG
		R:CATGATGGAGTTGTAGGTGGTCT

## Data Availability

The authors declare that all data supporting the conclusions of this study are available within the article.

## Supplementary Materials

The following supporting information can be downloaded at https://www.mdpi.com/article/10.3390/ani15142134/s1, Table S1: Statistics of the sequencing data; Table S2: Comparison of sequencing data with the reference genome.
