# Supplementary material for: Copper Pyrithione Induces Hepatopancreatic Apoptosis and Metabolic Disruption in Litopenaeus vannamei: Integrated Transcriptomic, Metabolomic, and Histopathological Analysis
Source: Animals (Basel). 2025 Jul 18;15(14):2134. doi: 10.3390/ani15142134 (PMC12291759; doi:10.3390/ani15142134)
Supplement: Supplementary file 1 [file animals-15-02134-s001.zip › animals-3726569-supplementary.pdf]

Table S1 Statistics of the sequencing data

Sample	Raw Reads	Clean Reads	Clean Bases（Gb）	Error Rate （%）	Q20（%）	Q30（%）	GC Content （%）
0 h	50949540	46426946	6.96	0.03	97.54	93.36	46.99
0 h	53784224	48429534	7.26	0.03	97.62	93.56	47.46
0 h	50983654	48469084	7.27	0.03	97.59	93.47	49.11
3 h	46816238	44108454	6.62	0.03	97.61	93.55	50.81
3 h	45654262	43251964	6.49	0.03	97.58	93.43	50.42
3 h	48270522	45696102	6.85	0.03	97.54	93.34	49.83
48 h	77722062	75311714	11.3	0.03	97.56	93.19	47.6
48 h	44652440	42221240	6.33	0.03	97.53	93.23	47.05
48 h	47687464	44742428	6.71	0.03	97.46	93.17	47.26

Table S2 Comparison of sequencing data with the reference genome

Sample	Total Reads	Reads mapped（%）	Unique mapped（%）	Multi mapped（%）	'+' mapped （%）	'-'mapped （%）
0 h	46426946	89.32	73.44	15.88	36.77	36.66
0 h	48429534	89.56	73.16	16.40	36.65	36.51
0 h	48469084	89.11	74.86	14.25	37.5	37.36
3 h	44108454	89.24	71.32	17.92	35.7	35.62
3 h	43251964	89.48	70.01	19.47	35.04	34.98
3 h	45696102	89.53	71.17	18.36	35.62	35.55
48 h	75311714	89.44	74.47	14.96	37.32	37.15
48 h	42221240	89.97	74.53	15.44	37.33	37.2
48 h	44742428	89.56	74.11	15.44	37.14	36.97
